# What Are the Experiences of Emotional Labor and Workplace Violence that Are More Harmful to Health in Korean Workforce?

**DOI:** 10.3390/ijerph17218019

**Published:** 2020-10-30

**Authors:** Won Ju Hwang, Hye Kyung Yang, Ji Hye Kim

**Affiliations:** 1 College of Nursing Science, Kyung Hee University, Seoul 02447, Korea; joabada@khu.ac.kr; 2 Research Institute of Korean Medicine Policy, Daejeon 34054, Korea; jihyekim1217@gmail.com

**Keywords:** workplace violence, emotional labor, job-related depression, health status, Korean Working Conditions Survey

## Abstract

(1) Background: This study aims to investigate the impact of emotional labor and workplace violence on sleep disturbance, depression, and health status in workers. (2) Methods: Data from 34,742 participants of the 2011–2014 Korean Working Conditions Survey were included in this study. We compared the incidence of sleep disturbance, depression, and health status according to emotional labor and workplace violence and used logistic regression to analyze factors that affect health status. (3) Results: Emotional laborers were more likely to experience sleep disturbance, depression and anxiety, and muscle pain. Workers who have experienced workplace violence were more likely to experience depression and anxiety, abdominal pain, and sleep disturbance. (4) Conclusion: Emotional labor and workplace violence have a grave impact on physical and mental health, with particularly greater effects on mental health. In addition, workplace violence has a greater health impact than emotional labor. The findings of this study suggest the need to implement programs that stabilize and heal workers who have experienced emotional labor and to enforce regulations and policies to protect workers from verbal and physical abuse.

## 1. Introduction

### 1.1. Background

Since the late 20th century, societies worldwide have experienced a rapid shift from a manufacturing economy to a service economy [1]. Korea’s industrial structure and labor are also following this trend, and according to a Korean statistics report, the service sector accounted for 58.3% of 2017 gross domestic product (GDP) and service-related workers accounted for 78.3% [2]. Although the employment rate has risen with the expansion of the service industry, bolstering competitiveness has consistently been a major target, and demands for customer-oriented, high-quality service production have risen [3]. Workers in the service industry are not only involved in emotional labor during work, but are also increasingly placed at risk of exposure to verbal or physical violence from customers. Their resulting physical and mental health conditions have surfaced as important societal issues. 

Emotional labor refers to labor that requires one to display emotions desired by the organization, irrelevant to actual emotions, when dealing with people [4]. A Korean study estimated that an approximately 7.4 million people (out of the economically active population of 17.70 million) are emotional workers, which accounts for 41.8% of wage workers [5]. Hochschild first introduced emotional labor in her book, *The Managed Heart*, and defined it as “the management of feelings to create a publicly observable facial and bodily display” [6]. Enterprises or organizations not only require laborers to abide by their display rule, but also want them to internalize the rule. In a work environment where workers must continuously manage their emotions and are therefore tense, workers experience various mental health conditions, such as anxiety, depression [7,8], and burnout [9], as well as diverse physical health conditions [10]. 

Workplace violence is another societal problem that must be addressed. It refers to assault, threat, bodily harm, injury, and all behaviors, incidents, and actions that deviate from reasonable action that occurs during or as a direct outcome of work [11]. Different types of workplace violence include assault or attack, threat, abuse, harassment, sexual harassment, and bullying/mobbing. Workplace violence can be divided according to the perpetrator into internal workplace violence inflicted by superiors or coworkers and external workplace violence inflicted by third parties, such as customers, consumers, or patients. Although few studies have examined the current state of workplace violence in Korea, it is estimated that about 4.19 million workers are exposed to significant mental or sexual violence from customers [12]. Workplace violence encompasses a broad spectrum from harassment to murder, and it has a physically and mentally detrimental impact on workers, including mental pain, depression [13], sleep disturbance [14], increased musculoskeletal symptoms [15], changes in subjective health status [16], and reduced self-esteem and job satisfaction, thereby increasing turnover, deteriorating service quality, and lowering productivity [17].

In the field of industrial and occupational health, studies have investigated the health impact of emotional labor and workplace violence and show that these two factors are significantly correlated [18]. Rupp and Spencer reported that occupations that require high emotional labor more frequently involve interaction with violent customers, unfair treatment, and greater mental and physical fatigue [19]. However, despite the fact that emotional labor and workplace violence do not always occur concurrently, they are sometimes used interchangeably. It is often considered natural for emotional laborers to be exposed to workplace violence in the Korean society [12], so it is necessary to examine the health impact of both factors together. Thus, in contrast to most previous studies that only utilized data from a single year [20,21], this study utilized data about workers’ individual factors, working conditions, and psychosocial work environment from the third and fourth Korean Working Conditions Surveys (KWCSs) to investigate the impact of emotional labor and workplace violence. 

### 1.2. Aim and Hypotheses 

This study investigated the impact of workers’ emotional labor and experience of workplace violence on their sleep disturbance, depression, and health status, and analyze the factors of work environment that predict sleep disturbance, depression, and health status in workers who have experienced emotional labor and workplace violence.

Hypotheses are as follows.

(a) Workers who have experienced workplace violence and emotional labor will experience physical health problems such as headache, eye strain, and abdominal pain more than those who do not.
(b) The scores of violence experience and emotional labor experience will be higher as factors influencing workers’ physical health.
(c) The scores of violence experience and emotional labor experience will be higher as factors affecting the mental health of workers.

## 2. Materials and Methods 

### 2.1. Study Design 

**Figure 1:**
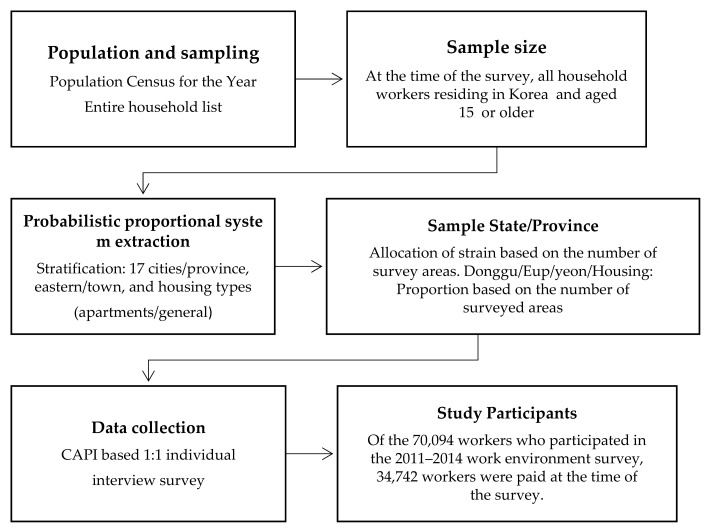
Sample Design Procedure of the Study.

This study uses a secondary data analysis of results from a descriptive survey (the 2nd–4th (2011–2014) KWCS raw data) to identify the impact of emotional labor and workplace violence on sleep disturbance, depression, and health status among workers. Sample design procedure of the study was depicted in Figure 1. 

### 2.2. Data Source and Study Participants 

The KWCS is published by Statistics Korea and the Korea Occupational Safety and Health Agency (KOSHA), and it is conducted on employed individuals aged 15 years or older nationwide. In this study, the participants from the 2nd–4th KWCS were enrolled. We obtained permission for using the 2011–2014 KWCS raw data from the Occupational Safety and Health Research Institute (OSHRI) for this study. We obtained the IRB exemption approval (KHSIRB-19-063(EA)). The KWCS is a nationally approved statistics dataset conducted in three-year intervals by the OSHRI per Article 18 of the Statistics Act. The KWCS is a survey conducted with employed individuals aged 15 years or older through individual interviews in the participants’ homes. The target population includes individuals living in a household in the Republic of Korea as of the time of the survey, and individuals who lived in households in apartment or ordinary enumeration districts as of the 2010 Population and Housing Census. Of them, this study enrolled self-employed/business owners, wage earners, unpaid family workers, individuals taking a temporary leave of absence from work, and other workers who worked in the past week (at the time of survey) for income. The survey population for this study comes from households in districts excluding islands, dormitories, special social facilities, hotels, and foreigner enumeration districts in the “Population and Housing Census.” The survey population was stratified into seven special and metropolitan cities and nine provinces, and the nine provinces were further stratified into dong and eup/myeon. 

In the sampling design, enumeration districts are the first sampling units and households are the second sampling units. The enumeration districts were sampled using probability proportional to size (PPS), where the sample is proportional to the number of households within an enumeration district. When hierarchical sampling was applied, the enumeration districts in each stratum were aligned according to their administrative zone number, and PPS was applied such that the sample could geologically represent the respective strata. The surveys were conducted through personal visits to the sample households by members of a professional survey and research group, and data were collected via computer-assisted personal interviewing (CAPI) using a tablet PC (Statistics Korea, 2014) [22]. Of the 70,094 workers who participated in the 2011–2014 KWCS, 34,742 of them were wage workers who had worked in the past week for income and were enrolled in this study, excluding self-employed/business owners, wage earners, unpaid family workers, individuals taking a temporary leave of absence from work, and other workers.

### 2.3. Definitions and Measurement of Variables 

The Working Conditions Survey (WCS) is utilized for making safety and health policies in European countries and the European Union (EU), and the 2011–2014 KWCS, which was used in this study, is a survey that benchmarked the EU’s WCS, using nearly identical items and methods. Of these items, we used work environment and psychosocial factors as our controlled variables, with reference to previous literature, in order to analyze the impact of emotional labor and workplace violence on depression, sleep, and health status.

#### 2.3.1. Emotional Labor

Emotional labor refers to customer service labor that involves a display of emotions and expressions demanded by the employer even amid situations in which they feel good, sad, or angry [23]. In general, flight attendants, call center employees, hotel and restaurant employees, and sales workers in department and discount stores are regarded as emotional laborers. However, in recent years, workers in many other job types have been found to do emotional labor during work or in regard to their relationships with coworkers and superiors [24]. 

In this study, emotional labor was assessed using the KWCS item asking about “work situations.” Among people who chose “I am emotionally involved in work” or “I have to hide my emotions during work”, those who answered “always true”, “mostly true”, and “occasionally true” were classified as emotional laborers, while those who answered “rarely true”, “never true”, or “not applicable” were classified as non-emotional laborers. 

#### 2.3.2. Workplace Violence 

Workplace violence refers to any act of abuse, threat, or assault in a work-related situation, including commuting to work, that clearly influences one’s safety, health, and wellbeing [25]. Violence encompasses a broad spectrum, from harassment to murder [26], and physical violence, mental violence, abuse, bullying, and sexual harassment are included in this spectrum. 

In this study, workplace violence was assessed using yes-or-no questions asking about whether respondents have been physically abused, bullied, harassed, or sexually harassed during work by a coworker, superior, or junior colleague. Those who answered “yes” to at least one of these questions were considered to have experienced workplace violence. 

#### 2.3.3. Sleep Disturbance 

Sleep disturbance refers to when one’s sleep needs cannot be satisfied, and includes changes in sleep duration, regularity, or time that results in insomnia or daytime fatigue [27], which hinders desired lifestyle or causes discomfort [28].

In this study, sleep disturbance was assessed using the question “Did you have any health problems in the past 12 months?” and a yes-or-no question asking about history of insomnia or sleep disturbance in this period. Those who answered “yes” were considered to have experienced sleep disturbance. 

#### 2.3.4. Depression

Depression refers to a mood disorder characterized by sad and miserable feeling, worries, gloom, helplessness, and feeling of unworthiness [29]. In this study, depression was assessed using the question “Did you have any health problems in the past 12 months?” and yes-or-no question asking about the history of depression or anxiety disorder. Those who answered “yes” were considered to have experienced depression. 

#### 2.3.5. Health Status

Workers’ health can be understood based on health diagnosis from a physician or workers’ self-rated health. However, it is highly difficult to examine objective health status based on physician’s diagnosis in large-scale epidemiological studies on workers. Thus, many studies measure subjective health status. Subjective health status refers to one’s perceived health status, and it has been identified as a sensitive indicator of one’ health status [30]. In this study, health outcomes were measured as perceived physical and mental health status [31]. 

Physical health status was assessed based on “yes” responses to the questions asking about the history of work-related musculoskeletal pain (low back pain, arm muscle pain, and leg muscle pain), headache, eye fatigue, and abdominal pain in the past 12 months. Mental health status was assessed based on “yes” responses to the question asking about the history of depression or anxiety disorder, general fatigue, insomnia, or sleep disturbance in the past 12 months.

### 2.4. Statistics Analysis

To examine the impact of emotional labor and workplace violence on depression, sleep, and health status in wage earners in Korea, we analyzed the 2011–2014 KWCS data as follows: The differences in workers’ emotional labor and workplace violence according to demographic characteristics and work environments were analyzed with χ^2^ test or *t*-test. With reference to previous literature and variables measured in our data, gender, age, education level, wage, and job group were measured as sociodemographic variables, and job position, weekly work hours, work type (public, private), workplace size (number of employees), length of current employment in years, number of days of night overtime per month, and form of work were analyzed as work environment variables. In addition, the influence of emotional labor and workplace violence on general health status (subjective), depression or anxiety disorder, and insomnia or sleep disturbance was statistically analyzed using logistic regression. Sociodemographic and work environment variables that may influence the dependent variables (general health status, depression or anxiety disorder, and insomnia or sleep disturbance) were entered as independent variables in each analysis to control for them. All analyses were performed using the STATA v.13.1 software (Stata Corporation, College Station, TX, USA).

### 2.5. Ethical Consideration

This study used data from the 2011–2014 Korean Working Conditions Survey, the survey of the Occupational Safety and Health Research Institute (OSHRI). Data were collected on employees older than 15 years of age living in 16 cities or provinces in the South Korea. Trained interviewers collected data from each respondent after the appropriate IRB approval. The KWCS were nationally representative interview surveys and included questions relating to workers’ socioeconomic data, workplace environment, and social and occupational health. Informed consent was obtained from all participants in the survey, and detailed information about the survey is available at the following websites: http://www.kosha.or.kr/jsp/kwcs/ for the KWCS. 

The analysis data of this study are data that the Korea Institute of Occupational Safety and Health has released to researchers. After the researcher joined the website of the Korea Institute of Occupational Safety and Health, the name of the data user, affiliation, contact information, purpose of use, etc. were revealed, and public data was requested. The Korea Institute of Occupational Safety and Health reviewed the use of data and approved the use of the data and provided the raw data through e-mail. The received data did not contain any information that could identify the personal information of the subjects participating in the investigation. This study was approved for deliberation exemption by the Investigational Review Board of the institution to which the author belongs by analyzing secondary data (KHSIRB-19-063(EA)). The KWCS data does not include personal identification information that can be estimated by the nature of the data. To protect privacy, the received data did not contain any information that would enable identification of any participating individuals”.

## 3. Results 

### 3.1. Variation in Emotional Labor and Workplace Violence According to Demographic Characteristics and Work Environment 

Participants’ general characteristics and work environment are shown in Table 1 and Table 2.

A total of 88.0% of the participants were men, and the mean age was 42.3 years. Among the participants, 47.6% were college graduates or higher, and 37.8% had a monthly income of less than two million KRW. A total of 24.7% of the participants were office workers, and 78.9% of wage earners were regular workers, with 38.9% working 40 h or less a week. While 86.2% of the workers worked in the private sector, 77.3% of the participants worked in a business with fewer than 50 employees. A total of 39.6% of the participants have worked less than five years at the current workplace, and 87.5% the participants did not work night overtime. 

In the emotional labor and non-emotional labor groups, the percentages of male workers were 88.5% and 86.9%, respectively, with a mean age of 41.7 years and 43.6 years, respectively, showing that the emotional labor group is statistically significantly younger (*p* ≤ 0.001). The most common education level and monthly income were college graduates or higher and less than two million KRW in both the emotional labor and non-emotional labor groups. In job category, office work was the most common in the emotional labor group (25.8%), with no difference between the two groups, and the percentages of sales and service workers were statistically significantly higher in the emotional labor group than in the non-emotional labor group (*p* ≤ 0.001). In terms of employment characteristics, the percentage of regular workers was higher in the emotional labor group, but there were no differences in form of employment between the two groups. The most common work hours was 40 h or less per week in both groups, while a higher percentage of the emotional labor group worked more than 52 h a week (32.7%). Regarding the number of employees and length of employment in the current workplace, there was a statistically higher percentage of regular workers who worked between 1–4 years in a small business with fewer than 50 employees in the emotional labor group than in the non-emotional labor group (*p* ≤ 0.001). While the greatest percentage of workers in both the emotional labor group and non-emotional labor group did not work night overtime, the percentage of workers who work night overtime was statistically higher in the emotional labor group (*p* ≤ 0.001).

In the workplace violence and non-workplace violence groups, the percentages of male workers were 84.9% and 88.2%, respectively, with a mean age of 41.1 years and 42.3 years, respectively. There was a statistically significantly difference in education level between the workplace violence and non-workplace violence groups, where 48.7% were high school graduates in the former and 48.0% were college graduates in the latter. The most common monthly income was less than two million KRW in both groups. The percentage of service workers was the highest (27.2%) in the workplace violence group, while the percentage of office workers was statistically significantly the highest (25.1%) in the non-workplace violence group (*p* ≤ 0.000). The greatest percentage of workers in the workplace violence group were service workers (27.2%), followed by office workers (16.4%) and sales workers (16.1%). In terms of form of employment, regular workers showed a higher rate of workplace violence, but there was no difference between the two groups. Regarding work hours, 43.3% of the participants in the workplace violence group worked more than 52 h a week, showing increased workplace violence rate with increasing work hours. On the other hand, 39.6% of the participants in the non-workplace violence group worked 40 h or less a week, showing that workplace violence rate is statistically significantly lower among those who work fewer hours (*p* ≤ 0.000). Regarding the type of workplace, number of employees, and length of employment at current workplace, the percentage of people who experienced workplace violence was significantly higher among those who work in the private sector, those who work in a small business with fewer than 50 employees, and those who worked between 1–4 years at the current workplace. While the greatest percentage of workers in both the workplace violence and non-workplace violence groups did not work night overtime, the percentage of workers who work night overtime was statistically higher in the workplace violence group (*p* ≤ 0.001).

### 3.2. Variation of Health Outcomes According to Emotional Labor and Workplace Violence 

Table 3 shows the variation in health outcomes according to emotional labor and workplace violence.

A total of 71.0% of the participants claimed to have good self-rated physical health, while 17.7% and 1.5% experienced headache or eye fatigue and abdominal pain, respectively. A total of 9.5% of the participants had taken a sick day for a health problem. In terms of mental health, 24.1% experienced general fatigue, and the rates of insomnia or sleep disturbance and that of depression or anxiety were 2.4% and 1.3%, respectively. 

Regarding self-rated physical health, 70.7% of the emotional labor group and 71.5% of the non-emotional labor group reported good health, showing a lower percentage in the emotional labor group. The rate of headache or eye fatigue was 19.0% and that of abdominal pain was 1.7% in the emotional labor group, showing higher rates compared to the non-emotional labor group. The percentage of participants who have taken a sick day for a health problem was statistically lower in the emotional labor group (7.0%) compared to the non-emotional labor group (*p* ≤ 0.000). 

Regarding mental health, the rate of depression or anxiety was higher in the emotional labor group (1.5%) than in the non-emotional labor group (0.8%), and the rate of general fatigue was statistically significantly higher in the emotional labor group (26.0%) than in the non-emotional labor group (19.4%) (*p* ≤ 0.001). The rate of anxiety or sleep disturbance was also statistically significantly higher in the emotional labor group (2.7%) than in the non-emotional labor group (*p* ≤ 0.000).

Regarding self-rated physical health, a high percentage of participants rated their health as good in both groups, but the percentage was lower in the workplace violence group (59.9%) than in the non-workplace violence group (71.6%). The rate of headache or eye fatigue (36.1%) and rate of abdominal pain (5.8%) was higher in the workplace violence group than in the non-workplace violence group. The percentage of participants who have taken a sick day for a health problem was statistically lower in the workplace violence group (22.8%) compared to the non-workplace violence group (*p* ≤ 0.000).

Regarding mental health, the rates of general fatigue (42.2%), anxiety or sleep disturbance (7.8%), and depression or anxiety (6.1%) were all statistically significantly higher in the workplace violence group than in the non-workplace violence group (*p* ≤ 0.001).

### 3.3. Impact of Emotional Labor and Workplace Violence on Physical Health Outcomes 

Table 4 shows the impact of emotional labor and workplace violence on physical health outcomes. Workers who experienced emotional labor were 1.39 times more likely to experience muscle pain (95% CI = 1.29–1.49), 1.35 times more likely to experience headache/eye fatigue (95% CI = 1.24–1.47), and 1.13 times more likely to experience abdominal pain ((95% CI = 0.86–1.48) compared to those who did not experience emotional laborers, all of which were statistically significant.

Workers who experienced workplace violence were 2.23 times more likely to experience muscle pain (95% CI = 1.98–2.51), 2.76 times more likely to experience headache/eye fatigue (95% CI = 2.43–3.12), and 3.77 times more likely to experience abdominal pain (95% CI = 2.84–5.00) compared to those who did not experience workplace violence, all of which were statistically significant.

Among the covariates controlled for in the logistic regression, gender, education level, wage, occupation, form of employment, work hours, emotional labor, and workplace violence were significantly associated with muscle pain. Gender, age, occupation, form of employment, work hours, emotional labor, and workplace violence were significantly associated with headache/eye fatigue. Wage and workplace violence were significantly associated with abdominal pain. 

### 3.4. Impact of Emotional Labor and Workplace Violence on Mental Health Outcomes 

Table 5 shows the impact of emotional labor and workplace violence on mental health outcomes. Workers who experienced emotional labor were 1.82 times more likely to experience depression/anxiety (95% CI = 1.33–2.49), 1.39 times more likely to experience general fatigue (95% CI = 1.28–1.50), and 1.91 times more likely to experience sleep disturbance (95% CI = 1.51–2.41) compared to those who did not experience emotional labor, all of which were statistically significant.

Workers who experienced workplace violence were 5.13 times more likely to experience depression/anxiety (95% CI = 3.87–6.79), 2.28 times more likely to experience general fatigue (95% CI = 2.02–2.56), and 3.32 times more likely to experience sleep disturbance (95% CI = 2.61–4.22) compared to those who did not experience workplace violence, all of which were statistically significant. 

Among the covariates controlled for in the logistic regression, gender, occupation, work hours, number of employees, emotional labor, and workplace violence were significantly associated with depression/anxiety. Age, education level, work hours, emotional labor, and workplace violence were significantly associated with general fatigue. Gender, education level, occupation, work hours, number of employees, emotional labor, and workplace violence were significantly associated with sleep disturbance.

## 4. Discussion 

This study investigated the status of emotional labor and workplace violence among Korean workers and the impact of emotional labor and workplace violence on physical and mental health among Korean workers using the data from the 2011–2014 KWCS conducted by the KOSHA. 

Workers who experienced emotional labor were 1.91 times more likely to experience sleep disturbance, 1.82 times more likely to experience depression or anxiety, 1.39 times more likely to experience muscle pain, 1.39 times more likely to experience general fatigue, 1.35 times more likely to experience headache or eye fatigue, and 1.13 times more likely to experience abdominal pain, all of which were statistically significant. Workers who experienced workplace violence were 5.13 times more likely to experience depression or anxiety, 3.77 times more likely to experience abdominal pain, 3.32 times more likely to experience sleep disturbance, 2.76 times more likely to experience headache or eye fatigue, 2.28 times more likely to experience general fatigue, and 2.23 times more likely to experience muscle pain, all of which were statistically significant.

We confirmed that emotional labor and workplace violence have a significant impact on all physical and mental health parameters, which is consistent with the findings of previous studies [20,32,33]. In particular, we found that workplace violence has a greater influence on health than emotional labor does. This suggests that workplace violence has a more adverse impact on health compared to emotional labor, with a particularly greater influence on abdominal pain (3.77 times) and depression and anxiety (5.13 times). These results are consistent with previous findings that workplace violence has a substantial impact on health [34,35], and our findings showed that workplace violence has a greater influence on health compared to emotional labor. 

Both emotional labor and workplace violence were found to have an adverse impact on health, with a greater impact on mental health than physical health. This is in line with the results of a previous study on the relationship between workplace violence and anxiety/depression [20,36] and a Danish study on the relationship between workplace violence and depression [37], both of which show that mental factors have more harmful effects on health. 

Our results are due to the mechanism in which emotional labor or workplace violence triggers physical health conditions by provoking negative mood, hindering concentration, and causing fear in addition to inducing mental health conditions such as depression, anxiety, general fatigue, and sleep disturbance. Anxiety and depression result from stress caused by emotional labor or workplace violence, and because increased anxiety and depression hinders work and diminishes the productivity of enterprise [38], they are significant in both the productivity of enterprise and workers’ health. 

Emotional labor and workplace violence tend to be considered inevitable in the sense that work involving customer service features frequent conflict and stress, and relationship conflicts are unavoidable in diverse interpersonal relationships within the workplace, including vertical relationships [39]. For this reason, emotional labor and workplace violence are not given proper attention. However, as found in this study, emotional labor and workplace violence have substantial effects on physical and mental health, and devising measures against them must be a priority. Workers who experience emotional labor and workplace violence experience a decrease in self-esteem, which in turn deteriorates the quality of service and affects their physical and mental health, thereby diminishing work focus, increasing turnover, and elevating social cost. For these reasons, creating measures against emotional labor and workplace violence are crucial not only for the individual, but also for society.

Health professionals or social workers who provide service to patients or clients are also vulnerable to the influence of burnout emotionally and physically because they are required to express their emotions and act appropriately [40,41]. In the health and social field, efforts should be made to improve the welfare of the working environment and reduce burnout [40].

Emotional workers must be recognized for their hard work and be properly compensated, and measures to treat workers respectfully and protect them from exposure to emotional labor and workplace violence need to be developed. Foster et al. [42] argued that violence prevention policies within the workplace are essential components of violence prevention programs. Campaigns that increase the awareness of the perils of workplace violence and ameliorate social culture, relevant education, and policies to foster a healthy workplace culture are needed.

In this study, it was found that women had a greater effect on physical and mental health through emotional labor and workplace violence than men did. This is consistent with the study by Stepanikova et al. (2020) the gender disparity affects women’s mental health [43] and a study by Harnois et al. (2018) that gender discrimination in women in the workplace causes health inequality was found [44]. Perceived gender discrimination is an important factor in women’s mental health, and cooperation is needed from a social perspective on many inequalities that contribute to women’s inappropriate treatment according to their gender [45].

In previous studies, the intensity of emotional labor and workplace violence were shown to increase with decreasing education level [46], temporary job positions, and larger enterprise size [20], which was contradicted in our study. However, considering that Park and Kim [47] and Kim [48] reported that violence victimization rate increased with increasing education level, we can surmise that the impact of education level on violence varies by occupation or work environment, which calls for further studies. 

Additionally, the relationship between social norms and expectations interact with perceptions and expression of workplace violence and emotional labor [49]. There may some factors specific to Korean society that differ from other environments. However, we could not examine the value in the study due to the data which are the similar variables to the European Union survey.

This study has a few limitations. First, the scope of emotional labor lacked precision, as emotional labor was defined as “work that directly deals with people other than colleagues, such as customers, passengers, students, and patients”, and thus excluded emotional labor involved in interpersonal relationships with colleagues and superiors. Second, we could not obtain data on the details of emotional labor and workplace violence incidents, and thus could not examine variations in health outcomes according to the degree of emotional labor and workplace violence exposure. Moreover, the role of moderator or mediator variables of emotional labor and work violence could not investigated due to data availability issue. Third, the impact on physical and mental health was measured based on self-reported data and not a physician’s diagnosis, which should be taken into consideration. Fourth, participants could have avoided giving accurate answers regarding emotional labor and workplace violence experiences that they did not want to disclose, which leaves the possibility of underestimation of the data.

Notwithstanding these limitations, this study sheds light on the influence of emotional labor and workplace violence on health in Korean workers. To the managers, employers, and when managing workers’ health, businesses should note that emotional labor and workplace violence could have serious effects on workers’ physical and mental health and develop a system to prevent them, protect the workers, and heal workers who have bene victimized.

## 5. Conclusions

Emotional labor and workplace violence are closely linked to workers’ health, and they are serious problems that adversely affect individuals as well as families, communities, countries, and firms [50].

This study found that emotional labor and workplace violence have a considerable influence on physical and mental health, with a particularly greater effect on mental health, and that workplace violence causes a greater health impact than emotional labor.

Our findings showed that emotional labor and workplace violence are statistically significantly associated with physical and mental health, highlighting the need for developing intervention programs that prevent emotional labor and workplace violence and protect workers’ health. Hence, it is crucial to implement programs that stabilize and heal workers who are tired from emotional labor and to enforce regulations and policies to protect workers from unfair verbal and physical abuse.

This study is significant in attempting to examine how emotional labor and workplace violence are associated with physical and mental health in Korean workers and in providing foundational data for developing prevention and protective measures pertaining to emotional labor and workplace violence for workers. However, further studies that consider various occupations and work environments are still needed. Furthermore, if possible, the role of moderator or mediator variables of emotional labor and work violence would be investigated.

Most studies on emotional labor and workplace violence in Korea have been focused on investigating the current status on these issues. Therefore, subsequent studies should develop and assess various emotional labor and workplace violence prevention programs, including policies, education, and campaigns, in consideration of diverse work environments and occupations.

## Figures and Tables

**Table 1:** Comparison of demographic characteristics.

Variables	Categories	Total	EL	Non-EL	x^2^ or t	*p*	WV	Non-WV	x^2^ or t	*p*
		N (%)	N (%)	N (%)			N (%)	N (%)		
Demographic										
Total		34,742 (100)	24,737 (71.2)	10.005 (28.8)			1892 (5.5)	32.850 (94.5)		
Gender	Male	30,588 (88.0)	21,894 (88.5)	8694 (86.9)	17.55	<0.001	1606 (84.9)	28,982 (88.2)	18.98	<0.001
	Female	4154 (12.0)	2843 (11.5)	1311 (13.1)			286 (15.1)	3868 (11.8)		
		N = 34,742								
Age	M ± SE	42.3 ± 11.6	41.7 ± 11.1	43.6 ± 12.6	279.95	<0.001	41.1 ± 11.5	42.3 ± 11.6	25.65	<0.001
	<30	4710 (13.6)	3408 (13.8)	1302 (13.0)			317 (16.8)	4393 (13.4)		
	30–39	10,180 (29.3)	7453 (30.1)	2727 (27.3)			562 (29.7)	9618 (29.3)		
	40–49	10,887 (31.3)	7997 (32.3)	2890 (28.9)			564 (29.8)	10.323 (31.4)		
	50–59	6220 (17.9)	4268 (17.2)	1952 (19.5)			335 (17.7)	5885 (17.9)		
	60>	2745 (7.9)	1611 (6.5)	1134 (11.3)			114 (6.0)	2631 (8.0)		
		N = 34,742								
Monthly income	<100	4365 (12.6)	2573 (10.7)	1792 (18.5)	380.71	<0.000	197 (10.6)	4168 (13.1)	134.31	<0.000
	100–199	12,765 (37.8)	9219 (38.3)	3536 (36.5)			905 (48.8)	11,860 (37.1)		
	200–299	9500 (28.1)	6997 (29.1)	2503 (25.8)			511 (27.6)	8989 (28.2)		
	300	7157 (21.2)	5292 (22.0)	1865 (19.2)			240 (13.0)	6917 (21.7)		
		N = 33,787								
Occupation	professional	2520 (7.3)	1798 (7.3)	722 (7.3)	805.05	<0.001	69 (3.7)	2451 (7.5)	217.80	<0.001
	senior manager	233 (0.7)	179 (0.7)	54 (0.6)			11 (0.6)	222 (0.7)		
	Clerical worker	8484 (24.7)	6312 (25.8)	2172 (22.0)			309 (16.4)	8175 (25.1)		
	sales	5060 (14.7)	4010 (16.4)	1050 (10.6)			303 (16.1)	4757 (14.6)		
	service	6561 (19.1)	4961 (20.3)	1600 (16.2)			511 (27.2)	6050 (18.6)		
	skilled workers	3962 (11.5)	2659 (10.9)	1303 (13.2)			180 (9.6)	3782 (11.6)		
	semi-skilled	2728 (7.9)	1815 (7.4)	913 (9.2)			219 (11.7)	2509 (7.7)		
	Non-skilled workers	4638 (13.5)	2642 (10.8)	1996 (20.2)			277 (14.7)	4361 (13.4)		
	Agricultural	206 (0.6)	120 (0.5)	86 (0.9)			0	206 (0.6)		
		N = 34,392								

Note: EL—Emotional Labor; WL—Workplace Violence; SE—Standard Error.

**Table 2:** Comparison of working conditions characteristics.

Variables	Categories	Total	EL	Non-EL	x^2^ or t	*p*	WV	Non-WV	x^2^ or t	*p*
		N (%)	N (%)	N (%)			N (%)	N (%)		
Working Condition										
Employment	Fixed	24,868 (78.9)	18,010 (80.9)	6858 (74.2)	192.15	<0.000	1273 (73.2)	23,595 (79.3)	42.46	<0.001
	Non-fixed	4515 (14.3)	2976 (13.4)	1539 (16.7)			295 (17.0)	4220 (14.2)		
	Part time	2127 (6.7)	1287 (5.8)	840 (9.1)			171 (9.8)	1956 (6.6)		
		N=31,510								
Working hours	≤40	13,512 (38.9)	8920 (36.1)	4592 (45.9)	323.93	<0.000	51.4 (27.2)	12,998 (39.6)	179.92	<0.001
	40–52	10,637 (30.6)	7733 (31.3)	2904 (29.0)			568 (29.5)	10,079 (30.7)		
	≥53	10,593 (30.5)	8084 (32.7)	2509 (25.1)			820 (43.3)	9773 (29.8)		
		N = 34,742								
Organizations	Private	29,745 (86.2)	21,410 (87.1)	8335 (84.0)	57.15	<0.000	1661 (88.0)	28,084 (86.1)	8.74	<0.001
	Public	4089 (11.9)	2728 (11.1)	1361 (13.7)			186 (9.9)	3903 (12.0)		
	Public-private	402 (1.2)	270 (1.1)	132 (1.3)			27 (1.4)	375 (1.2)		
	Non-profit	256 (0.7)	166 (0.7)	90 (0.9)			13 (0.7)	243 (0.8)		
		N = 34,492								
Number of Employment	≤50	26,093 (77.3)	18,902 (78.2)	7191 (75.1)	66.39	<0.000	1460 (79.4)	24,633 (77.2)	12.17	<0.000
	50–299	5301 (15.7)	3751 (15.5)	1550 (16.2)			287 (15.6)	5014 (15.7)		
	≥300	2362 (7.0)	1528 (6.3)	834 (8.7)			92 (5.0)	2270 (7.1)		
		N=33,756								
Working Period	≤1 year	4920 (14.2)	3087 (12.5)	1833 (18.3)	224.84	<0.000	309 (16.3)	4611 (14.0)	31.60	<0.001
	1–5 years	13,758 (39.6)	10,064 (40.7)	3694 (36.9)			815 (43.1)	12,943 (39.4)		
	5–10 years	7048 (20.3)	5214 (21.1)	1834 (18.3)			369 (19.5)	6679 (20.3)		
	≥10 years	9016 (25.9)	6372 (25.8)	2644 (26.4)			399 (21.1)	8617 (26.2)		
		N = 34,742								
Night shift	0 day	30,412 (87.5)	21,578 (87.2)	8834 (88.3)	39.92	<0.000	1376 (72.7)	29,036 (88.4)	473.24	<0.001
	≤7	1423 (4.1)	1101 (4.5)	322 (3.2)			208 (11.0)	1215 (3.7)		
	7–13	1428 (4.1)	1023 (4.1)	405 (4.1)			135 (7.1)	1293 (3.9)		
	14–20	880 (2.5)	587 (2.4)	293 (2.9)			78 (4.1)	802 (2.4)		
	≥21	599 (1.7)	448 (1.8)	151 (1.5)			95 (5.0)	504 (1.5)		
		N = 34,742								
Shift work	shift	26,788 (77.1)	19,314 (78.1)	7474 (74.7)	3.59	0.058	1233 (65.2)	25,555 (77.8)	161.50	<0.001
	Waiting	2949 (8.5)	2315 (9.4)	634 (6.4)			659 (34.8)	7295 (22.2)		
	Non-shift	3143 (9.1)	2192 (8.9)	951 (9.5)			293 (15.5)	2850 (8.7)		
		N = 34,742								

Note: EL—Emotional Labor; WL—Workplace Violence.

**Table 3:** Difference in physical and mental health outcomes according to emotional labor and workplace violence.

Variables	Categories		Total	EL	Non-EL	x^2^ or t	*p*	WV	Non-WV	x^2^ or t	*p*
			N (%)	N (%)	N (%)			N (%)	N (%)		
Physical health	Self-rated health	Well	24,549 (71.0)	17,416 (70.7)	7133 (71.5)	52.05	<0.001	1133 (59.9)	23,416 (71.6)	180.72	<0.001
		Generally good	8986 (26.0)	6547 (26.6)	2439 (24.5)			622 (32.9)	8364 (25.6)		
		Poor	1067 (3.1)	667 (2.7)	400 (4.0)			136 (7.2)	931 (2.9)		
			N = 34,602								
	Headache/Eye stain	Yes	6132 (17.7)	4679 (19.0)	1453 (14.6)	95.38	<0.001	683 (36.1)	5449 (16.7)	464.30	<0.001
		No	28,470 (82.3)	19,951 (81.0)	8519 (85.4)			1208 (63.9)	27,262 (83.3)		
			N = 34,602								
	Abdominal pain	Yes	534 (1.5)	407 (1.7)	127 (1.3)	6.71	<0.001	110 (5.8)	424 (1.3)	240.46	<0.001
		No	34,068 (98.5)	24,223 (98.3)	9845 (98.7)			1781 (94.2)	32,287 (98.7)		
			N = 34,602								
	Presenteeizm	Yes	3304 (9.5)	704 (7.0)	2600 (10.5)	99.91	<0.001	431 (22.8)	2873 (8.8)	409.45	<0.001
		No	31,438 (90.5)	9301 (93.0)	22,137 (89.5)			1461 (77.2)	29,977 (91.3)		
			N = 34,742								
Mental health	Depression/Anxiety Disorder	Yes	448 (1.3)	371 (1.5)	77 (0.8)	29.93	<0.001	116 (6.1)	332 (1.0)	366.61	<0.001
		No	34,154 (98.7)	24,259 (98.5)	9895 (99.2)			1775 (93.9)	32,379 (99.0)		
			N = 34,602								
	Overall fatigue	Yes	8345 (24.1)	6407 (26.0)	1938 (19.4)	167.86	<0.000	798 (42.2)	7547 (23.1)	357.41	<0.000
		No	26,257 (75.9)	18,223 (74.0)	8034 (80.6)			1093 (57.8)	25,164 (76.9)		
			N = 34,602								
	Insomnia/ Sleep disturbance	Yes	828 (2.4)	655 (2.7)	173 (1.7)	25.97	<0.000	145 (7.7)	683 (2.1)	238.30	<0.000
		No	33,774 (97.6)	23,975 (97.3)	9799 (98.3)			1746 (92.3)	32,028 (97.9)		
			N = 34,602								

Note: EL—Emotional Labor; WL—Workplace Violence.

**Table 4:** Impact of Emotional Labor and Workplace Violence in Physical Health Outcome.

Variables	Categories	Musculoskeletal Pain	Headache/ Eye Pain	Abdominal Pain
		(*n* = 21,157)	(*n* = 21,157)	(*n* = 21,157)
		OR (95% CI)	OR (95% CI)	OR (95% CI)
Sex	Male Female	1.00 1.18 (1.07–1.30)	1.00 1.13 (1.00–1.27)	1.00 1.04 (0.75–1.45)
Age(year)		1.01 (1.01–1.01)	1.00 (1.00–1.01)	0.99 (0.98–1.00)
Education	≤Middle school High school≥ College	1.00 0.78 (0.70–0.87) 0.52 (0.46–0.59)	1.00 0.92 (0.80–1.05) 0.99 (0.85–1.15)	1.00 0.74 (0.51–1.07) 0.68 [0.44–1.05)
Monthly Income	<100 100–199 200–299 ≥300	1.00 0.85 (0.76–0.95) 0.89 (0.79–1.01) 0.80 (0.70–0.92)	1.00 0.90 (0.78–1.03) 0.98 (0.84–1.14) 1.04 (0.88–1.23)	1.00 0.51 (0.35–0.73) 0.48 (0.32–0.73) 0.38 (0.23–0.63)
Occupation	Service or sales Non-service	0.85 (0.79–0.92) 1.00	0.62 (0.57–0.68) 1.00	0.82 (0.63–1.07) 1.00
Employment status	Fixed Non-fixed Others	1.00 0.95 (0.86–1.04) 1.85 1.63–2.09)	1.00 0.85 (0.75–0.95) 0.94 (0.80–1.10)	1.00 0.78 (0.55–1.11) 0.73 (0.46–1.17)
Working hours	≤40	1.01 (1.01–1.02)	1.00 [1.00–1.01)	1.00 [0.99–1.01)
Number of employment	<50 50–300 ≥300	1.00 0.94 (0.86–1.03) 0.90 (0.79–1.03)	1.00 1.05 (0.95–1.17) 0.89 (0.76–1.03)	1.00 1.01 (0.72–1.40) 0.81 (0.46–1.43)
Emotional labor	Yes No	1.39 (1.29–1.49) 1.00	1.35 (1.24–1.47) 1.00	1.13 (0.86–1.48) 1.00
Workplace violence	Yes No	2.23 (1.98–2.51) 1.00	2.76 (2.43–3.12) 1.00	3.77 (2.84–5.00) 1.00

**Table 5:** Impact of Emotional Labor and Workplace Violence in Mental Health Outcome.

Variables	Categories	Depression/Anxiety Disorder	Overall Fatigue	Insomnia/Sleep Disturbance
		(*n* = 21,157)	(*n* = 21,157)	(*n* = 21,157)
		OR (95% CI)	OR (95% CI)	OR (95% CI)
Sex	Male Female	1.00 1.41(1.01–1.98)	1.00 0.98 (0.88–1.09)	1.00 1.52 (1.16–1.99)
Age		1.01 (1.00–1.02)	1.01 (1.00–1.01)	1.00 (0.99–1.01)
Education	≤Middle school High school ≥College	1.00 1.14 (0.74–1.77) 1.16 (0.70–1.91)	1.00 0.77 (0.69–0.86) 0.68 (0.59–0.77)	1.00 0.69 (0.50–0.95) 0.74 (0.52–1.07)
Income	≤100 100–199 200–299 ≥300	1.00 1.06 (0.68–1.66) 0.89 (0.54–1.47) 1.05 (0.60–1.81)	1.00 0.99 (0.88–1.12) 1.01 (0.88–1.15) 0.99 (0.85–1.15)	1.00 0.80 (0.57–1.13) 0.92 (0.63–1.34) 1.27 (0.85–1.92)
Occupation	Service or Sales Others	0.72 (0.53–0.97) 1.00	1.02 (0.95–1.11) 1.00	0.69 (0.54–0.87) 1.00
Employment status	Fixed Non-fixed Others	1.00 0.95 (0.65–1.38) 1.50 (0.96–2.33)	1.00 1.05 (0.95–1.17) 1.65 (1.45–1.88)	1.00 1.23 (0.93–1.63) 1.38 (0.95–2.01)
Working hours	≤40	1.01 (1.00–1.02)	1.01 [1.01–1.02)	1.02 [1.02–1.03)
Number of employees	≤50 50–300 ≥300	1.00 1.78 (1.30–2.43) 1.56 (0.94–2.57)	1.00 0.95 (0.86–1.04) 0.95 (0.82–1.10)	1.00 1.30 (1.01–1.66) 1.65 (1.19–2.27)
Emotional labor	Yes No	1.82 (1.33–2.49) 1.00	1.39 (1.28–1.50) 1.00	1.91 (1.51–2.41) 1.00
Workplace violence	Yes No	5.13 (3.87–6.79) 1.00	2.28 (2.02–2.56) 1.00	3.32 (2.61–4.22) 1.00
